# Parallel Evolution of Host-Attachment Proteins in Phage PP01 Populations Adapting to *Escherichia coli* O157:H7

**DOI:** 10.3390/ph11020060

**Published:** 2018-06-20

**Authors:** Chidiebere Akusobi, Benjamin K. Chan, Elizabeth S.C.P. Williams, John E. Wertz, Paul E. Turner

**Affiliations:** 1 Department of Immunology and Infectious Diseases, Harvard T.H. Chan School of Public Health, Boston, MA 02120, USA; chidi.akusobi@gmail.com; 2 Department of Ecology and Evolutionary Biology, Yale University, New Haven, CT 06511, USA; b.chan@yale.edu (B.K.C.); escpwilliams@gmail.com (E.S.C.P.W.); 3 Coli Genetic Stock Center, Department of Molecular, Cellular and Developmental Biology, Yale University, New Haven, CT 06511, USA; john.wertz@yale.edu

**Keywords:** antibiotic resistance, bacteriophage, *Escherichia coli*, experimental evolution, molecular evolution, phage therapy

## Abstract

The emergence of antibiotic resistance has sparked interest in phage therapy, which uses virulent phages as antibacterial agents. Bacteriophage PP01 has been studied for potential bio-control of *Escherichia coli* O157:H7, its natural host, but in the laboratory, PP01 can be inefficient at killing this bacterium. Thus, the goal of this study was to improve the therapeutic potential of PP01 through short-term experimental evolution. Four replicate populations of PP01 were serially passaged 21 times on non-evolving *E. coli* O157:H7 with the prediction that the evolved phage populations would adsorb faster and more efficiently kill the host bacteria. Dead-cell adsorption assays and in vitro killing assays confirmed that evolved viruses improved their adsorption ability on *E. coli* O157:H7, and adapted to kill host bacteria faster than the wildtype ancestor. Sequencing of candidate tail-fiber genes revealed that the phage populations evolved in parallel; the lineages shared two point mutations in *gp38* that encodes a host recognition protein, and surprisingly shared a ~600 bp deletion in *gp37* that encodes the distal tail fibers. In contrast, no mutations were observed in the *gp12* gene encoding PP01’s short tail fibers. We discuss the functional role of the observed mutations, including the possible adaptive role of the evolved deletions. This study demonstrates how experimental evolution can be used to select for viral traits that improve phage attack of an important bacterial pathogen, and that the molecular targets of selection include loci contributing to cell attachment and phage virulence.

## 1. Introduction

The increasing prevalence of antibiotic resistance coupled with the waning production of new drugs calls for the development of alternative treatments against bacterial pathogens [1,2,3,4,5,6,7,8,9,10]. One possibility is phage therapy, which uses virulent bacteriophages (phages) to treat bacterial infections. Soon after their discovery, phages were utilized as antibacterial treatments, especially in Eastern Europe [11]. Although largely ignored by Western medicine following the discovery of broad-spectrum antibiotics, a recent resurgence of interest in phage therapy has sparked studies that successfully demonstrate the efficacy of phages for treating infections in animal models [2,3,4,5,6,7]. Phages isolated from natural microbial communities are effective in treating experimental bacterial infections, and some studies have evolved naturally isolated phages to improve their infectivity, thereby improving prospects for efficient use in phage therapy [12,13,14,15]. Here, we used short-term in vitro experimental evolution of phage PP01 to increase its virulence (killing efficiency) on the bacterial pathogen *Escherichia coli* O157:H7.

*E. coli* O157:H7 is an important food-borne pathogen that causes hemorrhagic colitis, and in severe cases hemolytic-uric syndrome [1]. Phage PP01 has a dsDNA genome that is ~140 kb in size, and is related to phages in the family *Myoviridae*, for example T-even-like phages [1,9]. In 2002, phage PP01 was isolated alongside *E. coli* O157:H7 from swine stool samples in Japan [1]. Although phage PP01 growth potency on *E. coli* O157:H7 can vary (presumably due to subtle lab effects [15]), their co-isolation suggests that the bacteria constitute a natural host of the virus, whose binding specificity [16] could be harnessed in phage therapy targeting this pathogen [17,18,19,20]. The current study shows that phage PP01 grows inefficiently on *E. coli* O157:H7, affording the opportunity to harness short-term experimental evolution to study generalities of how the virus initially adapts to improve host binding as an important fitness component of lytic reproduction. We predicted that phage PP01 adsorption (attachment) would be a major initial target for selection because preliminary adsorption experiments revealed that PP01 poorly attached to *E. coli* O157:H7. Improved adsorption should greatly increase PP01’s rate of infection events [21], and thus we expected that mutations responsible for faster attachment should be strongly selected in evolving phage populations.

T-even phage adsorption is mediated by long tail fibers that recognize and bind host cellular receptors such as lipopolysaccharides (LPS) and outer membrane proteins [22,23,24,25,26]. In phage T2, gene *gp37* encodes the distal tail fibers, which on their tips contain the homologous host recognition and binding protein, encoded by *gp38* [22,25]. In phage PP01, protein Gp38 reversibly binds to the outer membrane protein C (OmpC) on *E. coli* O157:H7’s cell surface [26]. This event prompts the short tail fibers, encoded by *gp12*, to irreversibly bind to the LPS of *E. coli* O157:H7, allowing PP01 to inject its DNA into the host and begin the lytic infection cycle [8]. Thus, we predicted that the initial evolution of PP01 populations on *E. coli* O157:H7 would select for mutations in genes *gp37*, *gp38* and/or *gp12*, allowing the phage to adsorb faster to the bacteria through improvements in the reversible and irreversible binding processes.

We allowed four lineages of phage PP01 to evolve independently on *E. coli* O157:H7 via serial transfer in a short-term experimental evolution study comprising 21 passages on this host. Consistent with our prediction, we observed that all four evolved virus lineages improved their adsorption ability on *E. coli* O157:H7, and adapted to kill host bacteria faster than the wildtype ancestor. Sequencing of candidate genes revealed that the phage populations evolutionarily converged, evidenced by shared point mutations in *gp38*. Furthermore, all of the evolved populations unexpectedly underwent sizeable deletions (579–686 bp) in the same region of *gp37*. Comparisons between phage clones drawn from the same evolved population strongly suggested that the deletion mutation in *gp37* alone could result in improved host attachment. In contrast, no mutations were observed in *gp12*. Our study demonstrates how experimental evolution can be used to select for viral traits that improve phage attack of an important bacterial pathogen, and that the molecular targets of selection include loci contributing to cell attachment, and thereby impacting phage antibacterial virulence. Future studies investigating the therapeutic potential of naturally isolated phages should continue to capitalize on short-term experimental evolution designed to improve key viral traits useful for phage therapy.

## 2. Materials and Methods

### 2.1. Strains and Culture Conditions

*E. coli* O157:H7 bacteria with its two shiga toxin genes (*stx1*, *stx2*) inactivated (American Type Culture Collection #43888, Manassas, VA, USA) were cultured at 37 °C with shaking (185 rpm) in Luria Bertani (LB) broth with 10mM MgSO_4_ and 5 mM CaCl_2_. Phage PP01 was kindly provided by Y. Tanji (Tokyo Institute of Technology, Tokyo, Japan), and grown with bacteria in liquid culture for experimental evolution, and on bacterial lawns in agar overlays (0.4% top agar, 1.5% bottom agar) for plaque purification and enumeration of phage titers (plaque-forming units [pfu] per mL).

### 2.2. Experimental Evolution

Four replicate populations of phage PP01 (hereafter referred to as wildtype, wt) were propagated on non-evolving *E. coli* O157:H7 for 21 consecutive serial passages. At the start of the experiment, ~10^7^ pfu of a plaque-purified isolate of phage PP01 and 100 µL (~10^8^ cells) of overnight *E. coli* O157:H7 bacteria were added to four flasks containing 10 mL LB, creating an initial multiplicity of infection (MOI; ratio of phage particles to bacterial cells) of ~0.1. The mixtures were then incubated at 37 °C for 24 h with shaking. A 1 mL sample was taken from each culture, chloroformed, centrifuged (13,000 rpm for 1 min at 25 °C) to isolate a cell-free lysate of phage, and stored at 4 °C. A 10 µL aliquot of each lysate (~10^7^ pfu) was added to a new culture flask inoculated with 100 µL (~10^8^ cells) of naïve (frozen stock) stationary-phase bacteria cells. This process was repeated for 21 successive passages. The four evolved PP01 populations were denoted E1, E2, E3, and E4. We note that our experimental design purposefully focused on evolved changes in phage populations, and not on co-evolutionary changes between bacteria and phages, unlike prior studies [27,28].

Although prior work has observed generally robust growth (plaquing ability) of phage PP01 on *E. coli* O157:H7 [1], experiments in our laboratory with this phage isolate showed weaker growth (relatively small-sized plaques) for unknown reasons. However, such subtle lab-specific effects are not uncommon in microbiology [16]. Furthermore, sequencing revealed no difference in the *ompC* sequence of the *E. coli* O157:H7 host strain compared to the published sequence (data not shown), indicating that a change in *ompC* that could cause increased phage resistance cannot be responsible for weaker phage traits for the PP01 ancestor shown in our study.

### 2.3. Dead Cell Assay to Examine Phage Adsorption via Irreversible Binding

Phage adsorption was measured using a dead cell adsorption assay [17], which measures disappearance of phage particles as a result of irreversible attachment to bacteria that bind virus but do not support their growth; this process allows adsorption to be measured for extended time periods. Bacteria were killed by placing a 50 mL culture of stationary phase cells into a pre-heated 70 °C water bath for 1h. Dead cells were mixed 1:1 volumetrically with 50% glycerol in LB, and stored as 1mL aliquots at −80 °C. To measure attachment, dead cells (4 × 10^7^ cells/mL) and test phage (2 × 10^5^ pfu/mL) were mixed in LB to achieve an initial MOI = 0.01. Every 5 min for 20 min, a 100 µL sample was titered to track virus disappearance from the supernatant.

### 2.4. Bacterial Growth Curves

Growth of infected and uninfected bacteria was measured for 24 h in a 96-well plate using a microplate reader (Tecan, Männedorf, Switzerland, ES2000 Series), which tracked optical density at 600 nm (OD_600_). Similar high-throughput methods are increasingly used to compare growth estimates among bacterial strains and to infer growth ability of lytic phage strains that infect bacteria (e.g., [29,30]). In the current study, each well contained 200 µL of LB with bacteria, or bacteria mixed with a test phage strain at MOI ≈ 0.01.

### 2.5. Sequencing of PP01 Tail Fiber Genes

Depending on the intended analysis (see Results) genomic DNA was obtained from either a phage population or one or more plaque-purified clones drawn from a population, using the phenol-chloroform isoamyl alcohol extraction method. The DNA was ethanol precipitated, and re-suspended in 30 µL of sterile water. The genes *gp12*, *gp37*, and *gp38* were sequenced with appropriate primers. The PCR products were gel purified using a Qiagen Gel Purification Kit (Qiagen, Germantown, MD, USA) and sequenced at the DNA Analysis Facility on Science Hill at Yale University. Chromatograms were analyzed using the CLC DNA Workbench version 6.6.1 (CLC Bio, Katrinebjerg, Denmark). The amino acid sequence alignment of *gp38* was also completed with CLC DNA Workbench version 6.6.1.

## 3. Results

### 3.1. Increased Attachment in Evolved Phage Populations

**Figure 1:**
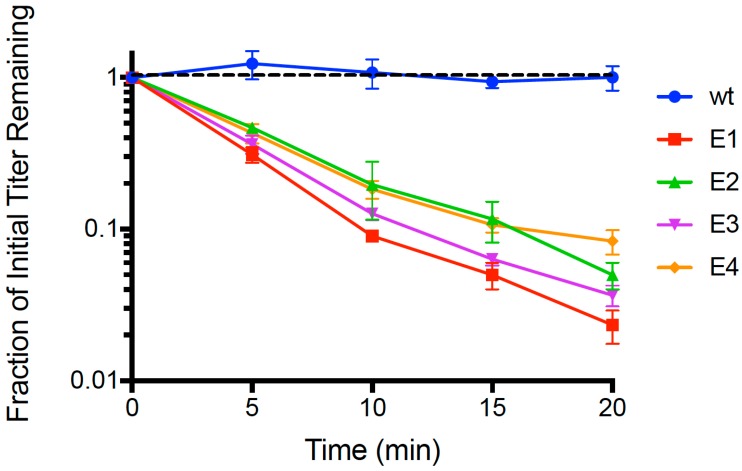
Adsorption of the wt ancestor (phage PP01) and each evolved phage population on dead *E. coli* O157:H7 cells (MOI = 0.01). Error bars represent 1 standard deviation (*n* = 3); error bars too small to be seen are omitted from the graph.

Following 21 passages of experimental evolution, we conducted repeated (*n* = 3) dead-cell attachment assays (see Methods) that measured adsorption ability of evolved populations (E1, E2, E3, E4) and the wildtype (wt) ancestor. We then estimated the mean fraction of unattached particles relative to initial titer (pfu/mL) in the supernatant, for each test phage; results are shown in Figure 1. After 20 min, we observed that the wt ancestor showed no measurable decrease in titer (−0.00241 ± 0.0027 SE; *p* > 0.05), indicating that any cell attachment by the wt in the time allowed was below the level of detection. In contrast, all four evolved lineages showed ≥90% mean disappearance (cell attachment) in this same time period. We used linear regression to estimate the rate of disappearance (negative slope) in each of the replicated population assays, and the grand mean rate of decline for each population. For all of the evolved populations, the negative slope of the grand mean regression was statistically significantly different than zero (*p* < 0.01). In addition, we used ANOVA to compare the attachment rates across all assays, and found that there was a significant difference in the grand mean regression slopes among the populations (*p* = 0.005). This outcome was consistent with E1 showing the most rapid observed mean decline (−0.0811 ± 0.0042 SE), which was significantly faster (*p* < 0.05) than the adsorption of E2 (−0.0646 ± 0.0038 SE), E3 (−0.0723 ± 0.0030 SE) and E4 (−0.0550 ± 0.0036 SE). Moreover, populations E2 and E3 did not significantly differ in grand mean slope (*p* = 0.156), but attached significantly faster than E4 (*p* < 0.05). In addition, we subjected the Figure 1 dataset to an additional analysis using multiple-comparisons ANOVA that compared each evolved population’s ‘fraction of initial titer remaining’ to that of wt at each time point (*t* = 5, 10, 15, 20 min); in all cases the evolved population titer was statistically significantly less than wt (*p* < 0.0001). From these analyses, we concluded that short-term evolution of PP01 populations on *E. coli* O157:H7 bacteria led to faster attachment of viruses to these host cells.

### 3.2. Improved Killing Efficiency of Evolved PP01 Populations on E. coli O157:H7

**Figure 2:**
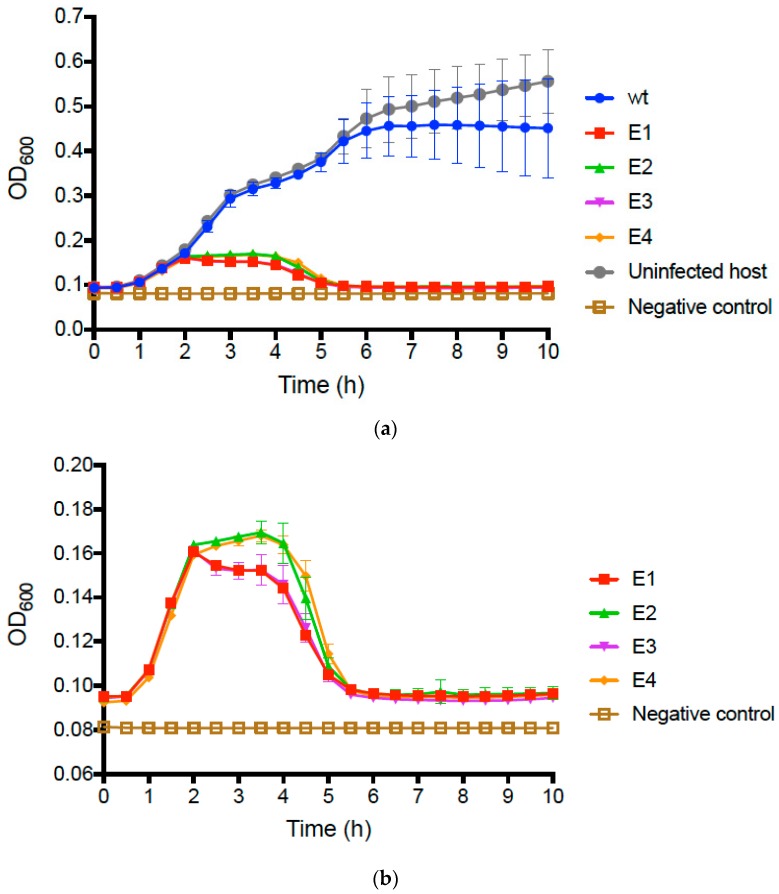
(**a**) Growth of *E. coli* O157:H7 cells infected with wt ancestor (phage PP01), and with each evolved phage population (E1–E4) at MOI = 0.01. Controls consisted of uninfected cells, and a negative control of media without added cells or phage. (**b**) Subset of the data in panel Figure 2a, presented at higher magnification to highlight negative effects of evolved phage populations on growth of *E. coli* O157:H7 cells. In both panels, error bars represent 1 standard deviation (*n* = 3); error bars too small to be seen are omitted from the graph.

We compared the growth of uninfected *E. coli* O157:H7 bacteria to that of bacteria infected with the wt PP01 ancestor by conducting repeated (*n* = 3) assays (see Methods) that tracked changes in bacterial densities (OD_600_ of growing cultures) over time. Results (Figure 2a) showed that infected and uninfected bacteria initially grew similarly, presenting mean OD_600_ ≈ 0.40 after 5 h and comparable growth trajectories. However, we then observed that growth of the wt PP01-infected bacteria plateaued at mean OD_600_ ≈ 0.45, whereas the uninfected bacteria continued to grow and reached mean OD_600_ ≈ 0.60 by 10 h (Figure 2a).

We then conducted identical assays comparing growth of bacteria infected with wt PP01 relative to bacteria infected by each evolved population. The data for the evolved populations qualitatively matched growth curves in assays for the uninfected and wt-infected bacteria during the first 2 h (Figure 2a); however, in the remaining 8 h of the assay, we observed dramatic differences between the assays containing evolved populations and those for the wt ancestor (Figure 2b). In particular, growth of bacteria infected by each of the evolved populations was highly similar in the latter stages of the assay; cells only achieved a maximum of OD_600_ ≈ 0.16, and after 5 h decreased to OD readings nearly as low as the negative controls containing no bacteria (Figure 2b). We performed multiple-comparisons ANOVA to compare the OD_600_ values of the test samples at 10 h. The differences between the wt, and each evolved population and the uninfected bacteria were statistically significant (in all cases, *p* ≤ 0.0001). Moreover, we determined that 10 h OD_600_ values did not statistically significantly differ for the evolved populations (*p* ≥ 0.573). These results confirmed that following experimental evolution, the phage populations generally evolved to kill the host bacteria far more efficiently than their wt ancestor.

### 3.3. Sequence Analysis of PP01 Tail Fiber Genes

We sequenced three genes that encoded tail fiber proteins in phage PP01: *gp12*, *gp37*, and *gp38*. The short tail fiber is encoded by *gp12* and the distal section of the long tail fiber is encoded by *gp37.* At the tip of the distal tail fiber is the host recognition protein encoded by *gp38*. Results of the dead-cell assays above suggested that evolved changes in phage populations included improvements in reversible and/or irreversible binding, indicating that mutations in either the short or long tail fibers could explain improved adsorption.

The NCBI consensus sequence (GenBank #AB180231) of *gp12* encoding the short tail fibers in phage PP01 shows a G/C polymorphism at bp 1196. Our sequencing results for *gp12* showed no differences between the ancestor and the evolved populations in this gene; all strains had a G at bp 1196 and no mutations at other sites in *gp12* (GenBank accession numbers pending). The lack of observed mutations in the short tail fibers indicated that irreversible attachment alone was not the target for selection in our experiment. The faster attachment rate of the evolved populations must then be attributed to an improvement in the reversible binding process, (mediated by *gp38*), and/or increased efficiency of the conformational changes in the phage as it deploys its short tail fibers prior to irreversible binding.

We sequenced *gp38* in the ancestor and evolved populations and compared these data to the NCBI consensus sequence (GenBank #AF349975). Results (GenBank accession numbers pending) revealed that the evolved populations all shared two non-synonymous polymorphisms: at bp 483 an A → C transversion led to a Q161H substitution, and at bp 620 a C → T transition caused a T207I substitution. In addition, each population showed a unique mutation in *gp38*. At bp 368, population E1 showed an A123E substitution, whereas population E4 showed an A123V substitution. Population E2 showed an N163H substitution, and population E3 had a Y159H substitution located nearby. The observed mutations in *gp38* suggested that suboptimal reversible binding was a target for strong selection in the experiment. In addition, the two mutations Q161H and T207I likely played key roles in the improved attachment since all four independently-evolved populations experienced these same convergent mutations.

To preliminarily examine the role of individual allele substitutions in improved adsorption, we chose to study one representative population from the four evolved lineages; we sequenced *gp38* in seven plaque-purified clones (GenBank accession numbers pending) randomly isolated from population E3, at the end of the study. These seven clones were abbreviated as E3a through E3g. The sequences of the seven E3 clones showed that the polymorphisms in the E3 population were due to the simultaneous presence of genotypes with the wt *gp38* allele (E3b, E3c, E3d, E3e) and genotypes with all three novel mutations in *gp38* (E3f, E3g). In addition, the sequencing revealed that clone E3a had the Q161H mutation and wildtype alleles at the other loci. The association of these mutations with improved binding is further examined below.

Lastly, we sequenced *gp37*, which encodes the distal long tail fiber. The wt ancestor’s *gp37* sequence did not differ from the published sequence (GenBank #AF349974). In contrast, all four evolved populations had deletions in *gp37* that varied in length from 579 bp to 686 bp, but were found in the same general region: 1980–2650 bp of *gp37* (GenBank accession numbers pending). Populations E1 and E3 shared an identical 579 bp deletion in *gp37* (2061–2640 bp). Population E2’s deletion was 620 bp (1987–2607 bp), and population E4 had a 686 bp deletion (1945–2631 bp). Gene *gp37* is 3300 bp long; thus, the observed deletions comprised ~20% of the gene. PCR probes for the deleted sequence were unable to detect the presence of full-length *gp37* in any of the evolved populations. We concluded that evolution on the *E. coli* O157:H7 host bacteria led to convergent evolution of deletions in *gp37*, and below we discuss how this outcome may relate to the evolution of improved attachment in the phage PP01 derivatives.

### 3.4. Mutations in gp37 and gp38 Associated with Adsorption Changes in Population E3

We sought preliminary evidence that allele changes in *gp37* and/or *gp38* played a role in improved attachment of phage PP01 on *E. coli* O157:H7. To do so, we focused on assays containing clones E3b and E3f that were isolated from evolved population E3. This comparison was meaningful because clone E3b contained a large deletion in gene *gp37* and the wildtype sequence in gene *gp38*, whereas clone E3f contained the identical large deletion in *gp37* and all three mutations in *gp38*.

**Figure 3:**
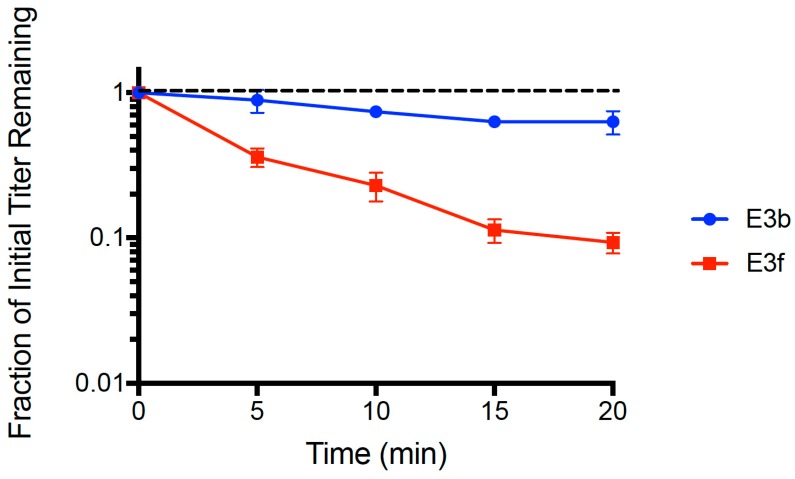
Adsorption of clones E3b and E3f on dead *E. coli* O157:H7 cells (MOI = 0.01). E3b has the wildtype gene *gp38* whereas E3f has fixed Y159H, Q161H and T207I mutations in *gp38*. Both clones contain an identical large deletion in gene *gp37*. The error bars represent 1 standard deviation.

We used the dead cell assay (see Methods) to compare binding abilities of clones E3b and E3f. Results (Figure 3) showed that both strains adsorbed faster than the wt ancestor (see Figure 1). A multiple comparisons ANOVA test was performed comparing the wt ancestor, E3b, and E3f for ‘fraction of initial titer remaining’ values at each time point (*t* = 5, 10, 15, 20 min) in the assay. The test showed statistically significant differences in the adsorption of all three phage populations at every time point (*p* < 0.005). The mean linear regression slopes for E3b (−0.0112 ± 0.0018 SE) and E3f (−0.0516 ± 0.0040 SE) were significantly less than the wt ancestor slope (−0.00241 ± 0.0027 SE, *p* < 0.01). Clone E3f adsorbed faster than clone E3b (*p* < 0.01), suggesting that one or more of the *gp38* mutations plays an important role in the improved binding. Importantly, clone E3b adsorbed faster (*p* < 0.05) than the wt ancestor indicating that improved binding of evolved PP01 populations could solely be due to the observed deletion in *gp37*. That is, because the E3b clone contained the deletion in *gp37* but was wildtype in *gp38*, our data indicated that the *gp37* deletion alone could explain much of the improvement in binding by evolved phage PP01 strains.

### 3.5. Amino Acid Sequence Alignment of gp38

**Figure 4:**
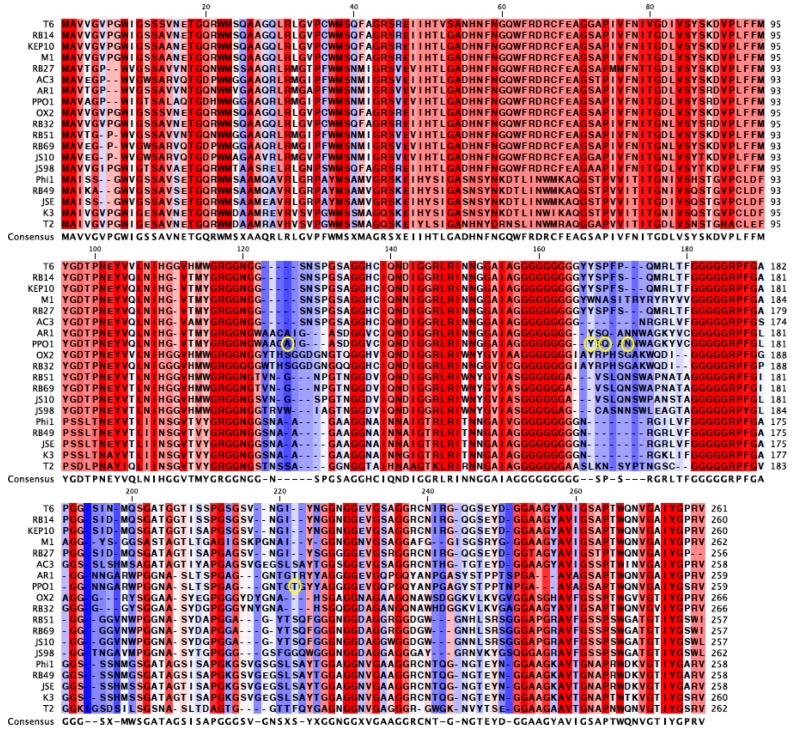
Gp38 amino acid sequence alignment of PP01 and its closest relatives. Amino acids in shades of red are highly conserved and amino acids in shades of blue are not conserved among the viruses. The shaded regions are the binding domains of the protein. The yellow circles indicate the mutated amino acids in the evolved populations.

An alignment of the *gp38* protein sequence of PP01 and its closest relatives revealed the highly conserved 120 amino acid N-terminus and 25 amino acid C-terminus regions of *gp38* (Figure 4) [31,32]. In Figure 4, the un-shaded regions signify those that are highly conserved amongst the viruses, whereas the shaded regions are not conserved. The alignment also revealed the conserved glycine repeats that flank the shaded hyper-variable regions of *gp38*, which serve as the protein’s binding domains. The mutations observed in the current study are circled in Figure 4. Three of the six mutations (Y159H, Q161H, N163H) were in the 2nd binding domain and were within seven amino acids of each other. These three mutations encode a histidine (Figure 4). Two mutations (A123E, A123V) were found in the 1st binding domain, and one mutation in the 4th domain (T207I). All of the observed mutations were located in Gp38’s binding domains, which corroborate the contribution of these mutations in increased adsorption of the evolved populations.

## 4. Discussion

We used short-term experimental evolution to select for improved binding performance of phage PP01 on *E. coli* O157:H7; this approach allowed us to examine whether experimental evolution led to increased infectivity of a candidate virus potentially useful in phage therapy. Wildtype PP01 was used to found four lineages independently evolved on non-co-evolving *E. coli* O157:H7 bacteria via 21 serial passages. We predicted that increased adsorption would be a major target for selection because viruses that more quickly bound to and infected host cells would on average produce progeny faster and maximize their fitness [21]. A dead cell assay was used to measure PP01 adsorption, where test PP01 populations were mixed with dead *E. coli* O157:H7 cells at an MOI of 0.01. The wt ancestor did not appreciably bind to the dead cells in the time allowed, whereas after 20 minutes the four evolved populations showed ~90% reduction in the supernatant (relatively increased binding) of the initial starting inoculum of virus particles. These results validated our hypothesis that cellular attachment would be a major target for selection in the short-term evolution experiment.

The dead cell assay measures irreversible binding, meaning that alterations in Gp12 and/or Gp38 could be responsible for an increase in adsorption. However, sequencing of *gp12* revealed that the ancestor and the evolved populations were identical in this gene for the short tail fiber, indicating that selection pressure to increase irreversible attachment by improving the ability of Gp12 to bind with the cell surface was not very strong in the time duration of our experiment. Moreover, these data suggest that complete irreversible attachment to *E. coli* O157:H7’s LPS might be a highly optimized step in PP01 adsorption, which does not have a high capacity for adaptive improvement over relatively brief evolutionary time.

The lack of mutations in *gp12* signified that the increase in attachment rate of the evolved populations likely involved improved reversible binding mediated by Gp38. Sequencing of *gp38* revealed all four evolved populations shared two polymorphisms (Q161H and T207I). The convergence of these mutations indicates that they play key roles in attachment. Each population also had a single unique polymorphism. Our hypothesis that the mutations in *gp38* should facilitate faster adsorption was supported by the dead cell assay performed with the E3b and E3f clones from population E3. Although both clones contained the *gp37* deletion (further discussed below), E3f had the Y159H, Q161H, and T207I mutations fixed in *gp38* while E3b had the wt *gp38.* Clone E3f attached faster to the dead host cells than E3b.

Further support that the *gp38* mutations function in enhancing adsorption stemmed from the locations of these mutations in *gp38’s* binding domains. Analysis of the mutations in *gp38* suggests that charged and hydrophobic amino acids allow the protein to bind to *E. coli* O157:H7 faster. Three of the six mutations in this study are to histidine (N163H in E2, Y159H in E3, and convergent mutation Q161H in all lineages), which has a positively charged side chain. Population E1’s A123E mutation encodes for glutamate, a negatively charged amino acid. These four mutations may allow Gp38 to better bind *E. coli* O157:H7 in a charge dependent manner. Furthermore, the three histidine mutations are in close proximity to one another in the 2nd binding domain, which suggests that this domain plays a key role in host recognition and may benefit from a more positively-charged binding site. In a past study, Morita et al. isolated PP01 host range mutants capable of infecting cells lacking OmpC. The host range mutants had mutations in *gp38* that were to positively charged amino acids arginine and histidine (Q161H, Q161R, W189R) [27]. This result suggests that the selection of positively charged amino acids in this region, especially at Q161, may increase adsorption by a mechanism other than direct binding to OmpC. Two of the polymorphisms in the evolved populations were to amino acids with hydrophobic side chains: A123V in population E3 and convergent mutation T207I. The A123V and T207I mutations suggest that Gp38 may better bind *E. coli* O157:H7 via hydrophobic interactions. The A123V mutation is particularly illuminating of the potential role of hydrophobic residues in *gp38* binding. Valine with its methyl group is marginally more hydrophobic than alanine, yet the A123V mutation was targeted by selection in population E4. Furthermore, the convergent T207I mutation converts tyrosine, a polar uncharged amino acid, to isoleucine, which has an uncharged hydrophobic side chain. Detailed knowledge of the specific residues that regulate attachment in *gp38’*s binding domains allows for the possibility of enhancing phage adsorption and altering host ranges in phage therapy candidates [33]. Current experiments in our lab concern the utility of genetic engineering of phages to increase their host attachment and specificity via *gp38* alterations.

**Figure 5:**
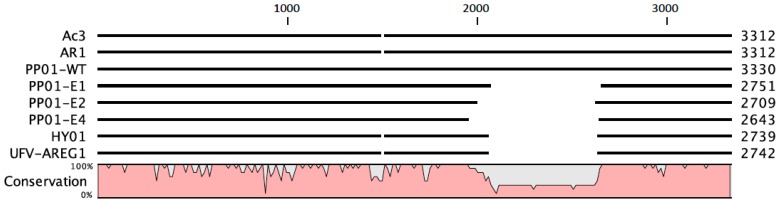
Nucleotide sequence alignments of closely related *gp37* distal tail fiber genes from four coliphages sometimes reveal deletions similar to those observed in the current study. PP01-WT is the wildtype sequence, while PP01-E1, -E2 and -E4 are evolved sequences containing the deletions described in the Results section. Phages AR1, PP01, HY01 and UFV-AREG1 can infect *E. coli* O157:H7, whereas phage Ac3 cannot.

At the onset of the experiment, we anticipated that the evolved populations would harbor mutations in *gp12* and *gp38* that facilitated faster attachment to the host. Surprisingly, *gp37* deletions in the 1980–2650 bp region arose early, (2nd or 3rd passage; data not shown) in all of the evolved populations and persisted until the 21st endpoint passage. The deletion mutation was unexpected because *gp37* encodes the distal long tail fibers and is not directly involved with adsorption to the host [34]. Nevertheless, the orientation of the proximal long tail fibers (gene *gp34*) to the baseplate determines short tail fiber triggering. It is possible that the shortened distal tail fiber causes more downward force to be applied at the baseplate by each bound long tail fiber. This could result in improved phage adsorption through a faster transition from reversible to irreversible binding [24]. T-even phages with faster contracting tail fibers are described as “trigger happy” and have been isolated in past experiments [22,23,25]. Further experiments are needed to confirm that our observations are explained by the “trigger happy” phenotype evolving in association with a mutation in the *gp37* distal tail fiber rather than in the baseplate genes [35]. Because phage PP01 seems to naturally infect *E. coli* O157:H7, one might ask: why has the phage not previously evolved this mechanism in the wild? To examine this question, we conducted a preliminary analysis using publicly available sequence data in GenBank. Figure 5 shows genetic data for *gp37* distal tail fiber genes in four coliphages with high sequence similarity to phage PP01, in comparison to phages in the current study. This comparison indicated that at least two other viruses (phages HY01 and UFV-AREG1) that could infect *E. coli* O157:H7 also possessed similar deletions as those observed when phage PP01 was subjected to experimental evolution; in contrast, phages AR1 and Ac3 do not show evidence of the *gp37* deletion. One possibility is that being “trigger happy” is not advantageous under some natural conditions, because this committed mechanism could lessen (or altogether prevent) phage detachment from sub-optimal host types, which are likely to be encountered in species-diverse natural communities of bacteria. In contrast, constant selection on a single host type in the laboratory may constitute a cost-free environment for evolving the “trigger happy” phenotype. This idea is speculative and warrants further investigation.

The *gp37* deletion evolutionarily preceded the mutations in *gp38* and was presumably the primary target of selection. The *gp38* mutations arose later in the experiment and were polymorphic in the final populations. Several factors could explain why the mutant alleles did not all fix in the final passages. First, the *gp38* mutations may have recently appeared and are in the process of fixing. Second, the large population size within each bottleneck (~10^7^ viruses) of the serial passage might have slowed the rate of fixation of beneficial mutations due to clonal interference, including mutations in *gp38* [36]. Lastly, phages with wt *gp38* may have beneficial mutations in other loci that confer an equal or greater fitness advantage than viruses with mutated *gp38.* Overall, we note that other loci may function in the adaptive changes allowing the PP01 derivatives in our study to better kill *E. coli* O157:H7, which can be addressed in future work involving single-step growth experiments and whole-genomics of the evolved populations. 

In addition to faster host adsorption, the evolved populations were also more virulent against *E. coli* O157:H7 than the wt ancestor. *E. coli* O157:H7 infected with wt PP01 was similar to the growth profile of the uninfected host and did not show a characteristic decline in density due to phage lysis over the time course of our growth assays. In contrast, the host infected with the evolved populations by 5h had OD_600_ measurements that nearly matched the negative control wells containing only growth medium. These results strongly suggest that the evolved PP01 populations would make better phage therapy candidates than wt PP01 because they not only attach to *E. coli* O157:H7 faster, but also are more virulent and exhibit higher bactericidal activity. Thus, after 21 passages of phage experimental evolution, we obtained evolved PP01 variants with greater therapeutic potential, which could be confirmed through additional studies, especially experimental mouse infections.

In the current study, we passaged phage PP01 populations on ‘static’ (non-coevolving) *E. coli* O157:H7 cultures, to rigorously investigate how the phage generally adapts to improve its infectivity on host bacteria. In the context of phage therapy, however, the host bacteria may not be static, and could instead co-evolve in response to the phage. Phage can exert selection pressure on the population of the target bacterial pathogen to evolve greater ability to resist phage attack; in response, the phage population can be selected to better overcome bacterial resistance mechanisms. We note that this co-evolutionary dynamic is possible, but not inevitable; rather, it depends on whether spontaneous mutations occurring in both the bacterial and phage populations provide the useful genetic variation needed to drive reciprocal evolutionary change. Another possibility is that phage evolution outpaces bacterial evolution (or vice versa), such that one interactor drives the other to extinction. Nevertheless, phage-bacteria co-evolutionary arms races have been documented previously through in vitro studies [15,37,38]; these co-evolutionary dynamics between phage and bacteria can be complex, with the possibility that populations of both interactors can evolve innovative defense strategies [39]. While the current study focused on evolved strategies that phages employ to better infect static host-bacteria targets, additional research could explore whether phage-bacteria coevolution leads to different phage adaptations than documented here.

Previous studies have examined the effectiveness of phage-therapy candidates—or of phage approved for emergency treatment—by relying on the spontaneous generation of highly virulent phage mutants or on isolates of virulent phage from natural sources [9,40,41,42]. In contrast, the current study suggests that experimental evolution may be an effective method to increase the killing potential of naturally isolated viruses targeted for phage therapy use, where parallel evolution among phage populations [43,44] can illuminate how phage-therapy candidates mechanistically improve killing ability on target bacterial pathogens. Future studies investigating the therapeutic potential of naturally isolated phage could benefit from using experimental evolution to improve key traits such as adsorption, virulence, and burst size before using phage to treat in vitro and in vivo experimental infections.
